# Effectiveness of the First Dose of BCG against Tuberculosis among HIV-Infected, Predominantly Immunodeficient Children

**DOI:** 10.1155/2015/275029

**Published:** 2015-06-29

**Authors:** Joaquim C. V. D. Van-Dunem, Laura C. Rodrigues, Luiz Claudio Arraes Alencar, Maria de Fátima Pessoa Militão-Albuquerque, Ricardo Arraes de Alencar Ximenes

**Affiliations:** ^1^Departamento de Pediatria, Universidade Agostinho Neto, Avenida Amilcar Cabral, s/n, Maianga, Luanda, Angola; ^2^Department of Epidemiology and Population Health, London School of Hygiene and Tropical Medicine, Keppel Street, London WC1E 7HT, UK; ^3^Departamento de Medicina Tropical, Universidade Federal de Pernambuco, Avenida Professor Moraes Rêgo, s/n, Bloco A do Hospital das Clínicas, Cidade Universitária, 50670-420 Recife, PE, Brazil; ^4^Pós-Graduação em Medicina Tropical, Universidade Federal de Pernambuco, Avenida Professor Moraes Rêgo, s/n, Bloco A do Hospital das Clínicas, Cidade Universitária, 50670-420 Recife, PE, Brazil; ^5^Departamento de Saúde Coletiva, CPqAM, FIOCRUZ, Avenida Professor Moraes Rêgo, s/n, Campus da UFPE, Cidade Universitária, 50670-420 Recife, PE, Brazil; ^6^Programa de Mestrado e Doutorado em Ciências da Saúde, UPE, Rua Arnóbio Marques, No. 310, Santo Amaro, 50100-130 Recife, PE, Brazil

## Abstract

The objective of this study was to estimate the protective effect of Bacille Calmette-Guérin (BCG) vaccine against tuberculosis among (predominantly immunodeficient) HIV-infected children in Angola. A hospital-based case-control study was conducted with 230 cases, children coinfected with tuberculosis, and 672 controls, HIV-infected children from the same hospital, aged 18 months to 13 years. The presence of a vaccination scar was taken as a proxy marker for BCG vaccination. The crude effectiveness was 8% (95% CI: −26 to 32) and the adjusted effectiveness was 30% (95% CI: −75 to 72). The present study suggests that BCG does not have a protective effect against tuberculosis among immunodeficient HIV-infected children. Since BCG is no longer given to HIV-infected children, the study may not be replicated. Accepting that these findings should be considered with caution, they are nonetheless likely to be the last estimate of BCG efficacy in a sufficiently powered study.

## 1. Introduction

Tuberculosis (TB) remains a public health problem worldwide. It is estimated that in 2012 there were 8.6 million incident cases, 13% of which included people living with HIV, with 1.3 million deaths (940,000 among HIV-negative individuals and 320,000 among HIV-positive) [1]. In Angola, the incidence of TB was 320 per 100000 inhabitants [2]; it was estimated that among adults aged 15 to 49 the prevalence rate of HIV was 2.4% [3].

The only licensed vaccine for the prevention of TB and leprosy is Bacille Calmette-Guerin (BCG) [4]. However, there has been much debate regarding the advantages and disadvantages of BCG since its use was first introduced. Key elements of the debate surrounding the effectiveness of BCG have included safety, loss of tuberculin sensitivity as a diagnostic tool, and, especially, the broad range of BCG effectiveness against TB, in 17 trials and 10 case-control studies, from no protection to 83% (95% CI: 58% to 93%) [5].

Available data have consistently suggested that BCG protects against severe forms of TB. A meta-analysis on the efficacy of BCG vaccination in newborns and infants in the prevention of TB demonstrated that BCG vaccine provided a protective effect of 71% against TB deaths and 64% against TB meningitis [5]. A further meta-analysis revealed a BCG protective effect of 86% against miliary or meningeal TB in randomized controlled trials and 75% in case-control studies [6]. The question of whether BCG strains (which evolved with different mutations along time) provide different degrees of protection against TB has been raised [7–9], but the epidemiological evidence is that strains variation does not explain variation in protection [10].

The AIDS pandemic ravaging sub-Saharan Africa has brought about increased challenges for the BCG vaccine, particularly with regard to its safety [11–15] and effectiveness. For countries with a high burden of TB, in 2004, World Health Organization recommended a single dose of BCG vaccine after birth, unless the child presented with symptomatic HIV infection [16]. When the study was being conducted, vaccination of HIV-infected children was carried out according to this recommendation. However, due to evidence that HIV-infected children vaccinated with BCG at birth, who later went on to develop AIDS, presented a higher risk of disseminated BCG disease, WHO changed its recommendation and children who are known to be HIV-infected, even asymptomatic, should no longer be immunized with the BCG vaccine [17].

In Angola, BCG has been administered since the 1970s. Vaccine is given at birth or on the first contact with health institutions. The strain of BCG vaccine used is BCG-Connaught (BCG vaccine, Sanofi-aventis, Quebec, Canada), administered with the manufacturer's recommended dose.

With the available studies, it is not possible to obtain definitive conclusions regarding the effectiveness of BCG in protecting against TB in HIV-infected, immunodeficient children and adults [18, 19].

The present study was proposed to evaluate the effectiveness of the BCG vaccine against TB in HIV-infected, predominantly immunodeficient children in a tertiary hospital in sub-Saharan Africa.

## 2. Patients and Methods

### 2.1. Study Population

The study population consisted of HIV-infected, predominantly immunodeficient children aged 18 months to 13 years treated at Hospital Pediátrico David Bernardino (HPDB) in Angola from January 2005 to December 2006.

### 2.2. Study Design and Definition of Cases and Controls

We conducted a hospital-based case-control study. All HIV-infected children, both in- and outpatients, were investigated. Cases included HIV-infected children diagnosed with TB according to WHO criteria for TB adapted for areas of high HIV prevalence [21] and classified as confirmed and probable.

Children were assigned for investigation of TB according to at least one of the following criteria for suspicion of TB: contact with confirmed or presumed TB cases; not regaining normal health after measles or whooping cough; fever and/or cough for more than 2 weeks after treatment (including antibiotics for common pathogens); weight loss/failure to gain weight/moderate to severe malnutrition; generalized lymphadenopathy and/or regional lymphadenitis; meningeal syndrome, thoracic spinal deformity; and changes on chest X-ray, which did not improve after antibiotic treatment for common agents.

*(1) Confirmed Diagnosis (One of the Criteria Listed)*

- Detection of the tuberculous bacilli in tissues or secretions by microscopy or culture (requires two positive samples by microscopic examination if fluid or pulmonary secretion).
- Identification of the bacillus TB as* Mycobacterium tuberculosis* in culture.

*(2) Probable Diagnosis (Probable Diagnosis, If at Least Three Points Are Verified)*

- History of contact with suspected or confirmed case of TB.
- Tuberculin skin test ≥ 5 mm.
- Chest X-ray findings suggestive of TB.
- Histological appearance suggestive of TB in biopsy material.
- Cytochemical examination of body fluids (pleural fluid, cerebrospinal fluid, and peritoneal fluid) compatible with TB.
- Favorable response to anti-TB treatment.

The selected cases represented all cases of tuberculosis in HIV-infected children during the study period, and controls were the consecutive HIV-positive children treated at and/or admitted to the same hospital but with no clinical evidence of TB at a ratio of 3 controls per case. Diagnosis of HIV infection was performed in accordance with the standards of the Angolan Ministry of Health [22] (see Supplementary Material available online at http://dx.doi.org/10.1155/2015/275029 for diagnostic criteria and exams).

### 2.3. Establishing Exposure and Procedures for Data Collection

BCG vaccination was identified by the presence of scar observed on the deltoid region of the left arm [22]. Results were coded and kept secret until completion of the exams, which would thus either allow inclusion as a case or control or exclusion. Identification was undertaken by nursing staff blinded to the outcome of the study. Interviews were performed using a standardized questionnaire. The interviewer, the personnel performing the diagnostic tests, and the three physicians who collected the clinical history and conducted the physical examinations were unaware of the main exposure and outcome.

Data were coded, checked, and double entered. At the end of the investigation, the researcher, unaware of the children's vaccination status, gathered together all information regarding the clinical history, physical examination, and complementary exams and allocated the children to the case group (confirmed or probable) or the control group, according to WHO criteria for TB (adapted for areas of high HIV prevalence) [21].

### 2.4. Sample Size

The estimated sample size was 225 cases and 675 controls, based on a ratio of three controls per case, assuming a protective effect of 36% [23], with an estimated coverage of 62%, [20] a power of 80%, and an alpha level of 0.05. We used the Epi Info module 6.04d (2001, CDC, Atlanta, GA).

### 2.5. Statistical Analysis

Analysis was performed using Epi Info 6.04d and SPSS 11.0 for Windows (SPSS Inc, Chicago, USA). Crude and adjusted odds ratios and their respective 95% confidence intervals were estimated. Vaccine effectiveness was estimated as (1 − OR) × 100 [24].

A logistic regression model was used considering TB (confirmed diagnosis, probable diagnosis, and overall assessment) as the dependent variable and scar as the main explanatory variable. The potentially confounding variables were grouped into blocks of variables: biological (age in 3 groups, gender), family structure (orphaned, sibling died of AIDS, and relationship to the head of the family), healthcare (where treated: hospital or outpatient clinic, vaccines registered in accordance with the expanded program on immunization (EPI), regularity of AIDS consultations, adherence to antiretroviral medication, and distance to health center), socioeconomic (head of family's education, employment status, family income, number of people living at household, and housing conditions), and clinical condition (mode of HIV transmission, history of TB and pneumonia, contact with TB, nutritional status, clinical category of HIV infection, and degree of immunodeficiency). Investigation of the confounding factors was conducted using the following steps:

- Confounding criteria: an OR ≥ 1.4 and/or *p* ≤ 0.20, in association with TB in the unvaccinated group and/or in association with exposure (scar) in the control group, in the univariate analysis; we adopted the cut-off point of OR > 1.4 with *p* ≤ 0.2 for we assumed that a variable that would reach these values in the association with exposure and disease would be likely to distort the association between exposure and disease.
- Adjustment of the variables within the same block. Those with *p* ≤ 0.10 were kept in the model.
- Adjustment of the OR of the association between BCG vaccine and TB for the variables of each block. The age variable remained in the model in all blocks (Table 3).

The effectiveness of BCG was first adjusted for the variables that met the criteria for confounding within each block. These variables were then included in the final regression model, and those with *p* ≤ 0.05 remained in the model (Table 3).

The sensitivity and specificity of BCG scar reading compared with the vaccination card, and information from parents or guardians as gold standards, in the same group of children, has been published elsewhere by the same authors [20]. Sensitivity ranged from 81.3% (95% CI: 78.0–84.2) to 91.6% (95% CI: 88.4–94.0) when the gold standards were, respectively, information from the adult responsible for the child and the vaccination card. Specificity ranged from 90.5% (95% CI: 81.6–95.5) to 94.1% (95% CI: 87.7–97.4) when the gold standards were, respectively, the vaccination card and information from the adult responsible for the child.

Crude and adjusted effectiveness of BCG vaccination was calculated based on the presence of BCG scar and on the vaccination card. To assess the validity of the BCG scar, only those children who presented both information from parents or guardians and vaccination cards were considered (502 children) [20]. However, in the present adjusted analysis of the protective effect, we selected all children with a vaccination card (541 children) without considering whether information from parents or guardians was available (Table 3).

Effectiveness of BCG was evaluated in relation to the pulmonary and extrapulmonary forms of TB (Table 5). We did not estimate separate efficacy for immunocompetent and immunodeficient children because of the small number of immunocompetent children.

## 3. Results

### 3.1. Selection of the Study Population

**Figure 1:**
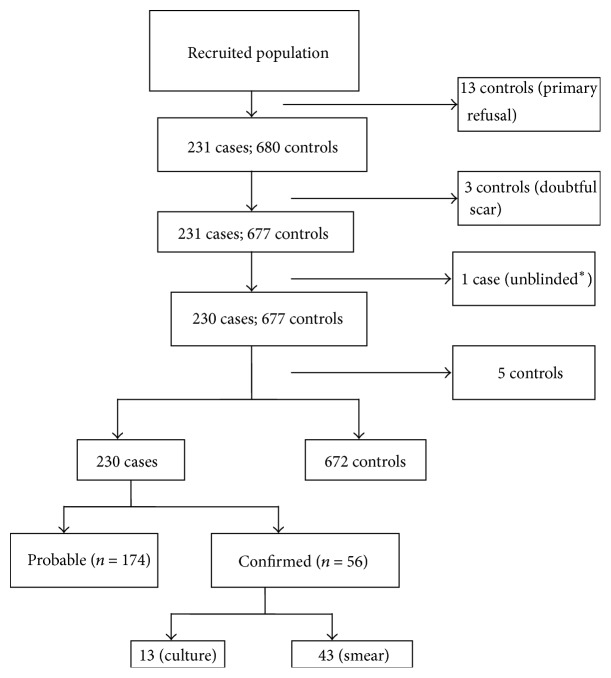
Selection of the study population. ^*∗*^Staff responsible for the X-ray reading discovered the diagnostic hypothesis before delivering the report.

A total of 231 cases were recruited. Of these, one was excluded because staff responsible for X-ray reading discovered the diagnostic hypothesis before delivering the report, which could have led to misclassification. A total of 230 cases and 672 controls were selected. Exclusion criteria and number of patients excluded are presented in Figure 1. Of the 230 cases, 156 (67.8%) were recruited from inpatients, while this percentage was 33% among the controls.

Of the 230 cases, 200 (86.9%) presented with pulmonary TB, of which 4 (2.0%) had severe pleural involvement. Extrapulmonary tuberculosis was identified in 30 cases, 13 of which (43%) presented with miliary TB; 5 (17%) presented with ganglionary TB; 5 presented with TB meningitis. Among cases, 15.2% were aged below 37 months, 41.3% were aged between 37 and 60 months, and 43.5% were aged 60 months and over; 52.6% were males. Of the 672 controls, 36.9% were aged below 37 months, 40.4% were aged between 37 and 60 months, and 22.6% were aged 60 months and over; 52.5% of controls were females. The median age of cases was 4.83 and 3.50 among the controls.

Table 1 presents a comparison of the main characteristics of cases and controls.

Table 2 presents the odds ratios and effectiveness of the association between BCG vaccination and TB according to different diagnostic categories. BCG scar was observed in 62.6% of the cases and in 64.4% of the controls. These results are similar to those observed when considering the categories of probable and confirmed diagnoses independently (Table 2).

In Table 3, we present the OR of the association between TB and BCG adjusted for the variables that remained in the multivariate model of each block of variables, where age remained in all models. The OR values ranged from 0.79 (when adjusted for healthcare variables) to 1.14 (when adjusted for clinical conditions variables). This association was not statistically significant in any of the blocks. Additionally, there was an overlap of the confidence intervals; hence there was no difference between them, and no wide confidence intervals were observed.

The median age of the children was 3.66 for those with a vaccination card and 3.91 for those with no vaccination card.

Crude and adjusted effectiveness of BCG vaccination based on the presence of BCG scar and on the vaccination card is demonstrated in Table 4. The crude and adjusted effectiveness, based on BCG scar when all children were considered, was 8% (95% CI: −26 to 32) and 30% (95% CI: −75 to 72), respectively. When only the subgroup of children with a vaccination card was estimated (*n* = 541), the crude effectiveness was 29% (95% CI: −14 to 56) if based on the vaccination card and 16% (95% CI: −32 to 47) if based on the scar (Table 4).

In an exploratory analysis, it was discovered that, among children with both the pulmonary and extrapulmonary forms of TB, the percentage of those vaccinated was similar to that observed amongst the controls. The effectiveness observed for pulmonary forms was close to the crude effectiveness for all children (Table 5).

The median ages of children with pulmonary and extrapulmonary forms were 4.83 and 4.96 years, respectively.

We compared probable and confirmed cases with controls in relation to clinical criteria (cough, sputum production, breathlessness, and fever), TST, history of contact with someone with TB, and variables related to HIV/AIDS (previous diagnosis of HIV, immunodeficiency, and clinical category of HIV (CDC)). The results showed that the group of confirmed and probable cases were very similar in relation to most of the above mentioned factors and that they differed from controls (data not shown), suggesting that the degree of misclassification in relation to the diagnostic (TB) was not large.

To evaluate the potential bias introduced by inclusion of TST ≥ 5 mm in the definition of probable TB we tested if there was an association between the induration size and BCG vaccination. In cases the frequency of TST ≥ 5 mm was high in vaccinated (96.53%) and in unvaccinated (91.86%) (*p* = 0.124), while in controls it was low in vaccinated (2.78%) and in unvaccinated (6.00%) (*p* = 0.377).

## 4. Discussion

This is the first study carried out in Angola to assess the protective effect of BCG in HIV-infected children. There has only ever been one other very small study conducted in Zambia, which estimated BCG efficacy in HIV-infected children [25]. Our findings suggest that the first dose of BCG has no protective effect against TB in these children.

Due to the lack of studies provided by the literature, the protective effect of BCG in HIV-infected individuals is still unclear. A case-control study was conducted with Colombian adults but with a small sample size (88 cases and 88 controls) [26], and although the protective effect against all forms of TB was 22%, the difference was not statistically significant (OR = 0.78, 95: 0.48 to 1.26). Other studies conducted with HIV-infected adults vaccinated with BCG in childhood have not indicated any protective effect against TB [26, 27].

As in the case of adults, there are very few studies on the protective effect of BCG in children. In Zambia, a hospital-based case-control study (116 cases and 154 controls) did not identify a protective effect of BCG against TB in HIV-infected children: OR = 1.0; 95% CI: 0.2–4.6 [25]. However, the wide CI suggests that the power of the study was not sufficient to detect a protective effect.

In the global general population, mostly assumed to be HIV-negative, BCG efficacy against pulmonary disease varies [28–32], ranging from 0 [27] to around 80% [30, 31]. Several explanations have been put forward concerning such variability, including the improper handling and administration of the vaccine, exposure to nontuberculous mycobacteria (NTM) in the periequatorial region, low immunogenicity of the vaccine, concomitant malnutrition, and other infectious diseases as well as a genetically determined low immune response to the vaccine [33, 34]. The most widely accepted explanation for the lack of protective effect against TB observed in regions up to 30° latitude of Ecuador has been a greater exposure to NTM [34–37]. These factors may also be responsible for the lack of protection encountered in our study; it is not clear how much the immunosuppression was related to the lack of protection.

BCG scar reading has been used as an indicator of vaccine status and became standard practice for assessing the protective effect of BCG in retrospective studies [38]. However, scars may be absent due to administrating lower doses of vaccine in childhood, the difficulty of injecting the entire amount of vaccine and the relatively weak local immune response in young children, or the probability that they may even disappear with time [39]. Furthermore, scar formation depends on the age of vaccination [40, 41], gender, [42] and the fact that other scars may mimic the BCG scar [43].

In the present study, the absence of scarring on those who had been vaccinated may have influenced the effectiveness, since the vaccinated children who did not develop a scar (if, for example, the vaccine no longer contained live BCG due to inadequate storage) may have been considered unvaccinated, thus generating a nondifferential misclassification, which could minimize the protective effect of the vaccine. A relevant subsidy to this discussion would be to discover the proportion of vaccinated children in Angola who develop and maintain scars. This proportion is around 60% in Sweden, 14 years after vaccination [44], and 98.9% in India, four years after vaccination [45]. Furthermore, development of protection after BCG vaccination cannot be assumed by the formation of a scar; there are no correlates of BCG protection.

To minimize misclassification bias, scar reading was performed by suitably trained personnel blinded to the outcome. Three infants, who presented with doubtful scars, were excluded. The sensitivity and specificity of BCG scar reading [46] were acceptable, suggesting no important classification bias. When only the vaccination card was considered as the gold standard, sensitivity was similar to that obtained in Brazil [47], India [45], and Malawi [48].

Another group of reasons that could explain the lack of protection is related to the different mechanisms of the disease [27]. It has been hypothesized that BCG has a high protective effect against newly acquired forms, the predominant form in our study considering the age of the children, and a low protective effect against endogenous reactivation [49]. On the other hand, it has been suggested that protection may only develop after three years of BCG vaccination [50]; a delay in development of protection would reduce the measured protection in a fraction of the children in our study as the time elapsed since vaccination would not be long enough. This study, through its characteristics, is unable to contribute to this debate.

A further set of reasons relates to the high prevalence of NTM via two proposed mechanisms, blocking and masking. BCG appears to be sensitive to the influence of preexisting immune responses to antigens shared by some strains of NTM [51]. Previous sensitization to NTM could block the development of protection. Alternatively, the higher prevalence of NTM would work as a “natural” vaccine against TB (but not leprosy), masking the measurement of a BCG protection that is still high, leading to variable efficacy against tuberculosis [52].

One point against prior exposure to NTM as a cause of BCG failure in this study is that, in Angola, BCG is given at birth or at “the first contact with the health center,” and, therefore, children would more likely be vaccinated before contact with NTM. However, interference from the MNT protective effect cannot be excluded: the age of vaccination was not recorded, but the percentage of births attended by health personnel may suggest “late contact with the health center,” hence allowing a previous contact with NTM. Another point is that the prevalence of NTM as well as the protective effect of BCG in HIV-negative children in Angola is unknown.

Finally, low efficacy may simply be a consequence of HIV infection and consequent immunodeficiency. In Zambia, efficacy was 59% in noninfected children, while it was 0% in infected children, although the study was small and the power was limited. In Argentina, a study on delayed complications of BCG vaccination in HIV-infected children reported a similar frequency of TB in both vaccinated and unvaccinated children [53]. Most cases and controls in our study were immunodeficient.

Lack of protection could potentially result from deficiencies in administrating the vaccine in addition to the above-mentioned factors. To clarify this point, it would be necessary to evaluate the quality of BCG vaccination, including the administration technique and the cold chain maintenance and management. This evaluation was beyond the scope of our study.

The difference in the unadjusted effectiveness between “all children” and the groups with vaccination scars may be related to a reduction of the sample size and selection bias introduced by this fact. Effectiveness was lower in those for whom evaluation was based on the vaccination scar compared to those for whom it was based only on the vaccination card, since it was more probable that those who had been vaccinated were in possession of a vaccination card.

The subgroup analysis (pulmonary and extrapulmonary) was just exploratory as the sample size was not estimated to make these comparisons. Effectiveness against the pulmonary form was close to that obtained when considering all forms of TB, reflecting the greater number of cases of pulmonary forms. The lack of a protective effect against extrapulmonary TB is contrary to the majority of studies reporting an effectiveness between 75% and 86% [6, 54]. Studies with the most consistently high protection have focused on severe extrapulmonary forms, meningeal and disseminated, which constituted around 60% of the extrapulmonary forms of the present study, possibly because of the exclusion of cases below 18 months. As this study was not designed to evaluate subgroups, the small number of cases limits the interpretation of this result.

Due to certain limitations of this study, results need to be interpreted with caution.

Refusal to participate, primary and secondary, though occurring only amongst controls, had little or no influence over the results because of low magnitude.

Owing to the difficulty of diagnosing pediatric TB, probable diagnosis was included as a category under study. Because it is based on symptoms and signs and X-ray findings, it may overlap with the findings of the HIV/AIDS, even in children without TB [55, 56], leading to an underestimation of the protective effect. However, the analysis, considering the two diagnostic categories (confirmed and probable), establishes the absence of a protective effect in immune deficient HIV-infected children, suggesting that classification bias, should there have been any, was minimal. Moreover the similarity between probable and confirmed cases of TB and their difference from controls in relation to clinical criteria, TST, history of contact with someone with TB, and HIV/AIDS related variables suggests that the degree of misclassification bias was not large.

The use of TST in the diagnosis of TB might lead to overrecruitment of vaccinated cases which might have led to an underestimation of efficacy. However, there was no association between BCG vaccination and the induration size of the TST either in cases or in controls. It is also possible that the controls included a number of children with unrecognized TB; however, if vaccine is not protective, they are likely to be not differentially distributed among controls with and without BCG scars, which, therefore, would not change the results.

The fact that this is a hospital-based study limits the generalization of results. However, HPDB is a Ministry of Health national reference in providing free treatment and care for children with HIV/AIDS. Some coinfected children may not have come to the hospital; it will not bias the results if, as we believe, it occurred similarly for vaccinated and unvaccinated cases. Controls were selected from the same hospital as the cases. As this is a reference hospital, cases and controls attending this service may have more severe forms of HIV/AIDS but it is likely that controls were subject to the same selection factors that influenced the cases to come to this particular hospital, and thus they would be comparable to the source population of the cases.

Since there is a lack of information regarding the precise moment when the BCG vaccine was given (although they were most likely not vaccinated at birth), we cannot exclude the fact that a cohort effect may have occurred.

## 5. Conclusions

In our study sample, we did not detect any protective effect of BCG vaccination against TB disease. BCG remains the only tool available for the prevention of severe forms of TB in HIV-uninfected children. Based on the evidence that children who were HIV-infected when vaccinated with BCG at birth were at increased risk of developing disseminated BCG disease, WHO, in 2007, recommended that HIV-infected children should not be vaccinated with BCG [17]. Following WHO recommendation (based on fears of adverse events and not on estimates of efficacy), no further studies will be conducted to explore potential explanations for this lack of protection. Although accepting that the findings of this study are difficult to interpret and should be considered cautiously, they are likely to be the last estimate of efficacy of BCG in a predominantly immunodeficient, HIV-infected children population in a sufficiently powered study. These data may also be useful as a historical comparison when novel TB vaccine candidates undergo clinical trials in HIV-infected infants.

## Supplementary Material

The supplementary material includes the diagnostic criteria for HIV infection in Angola and the techniques used in the study for the CD4 + T cells count, tuberculin test and Acid-Fast Bacilli (AFB) smear and culture.

## Figures and Tables

**Table 1:** Comparison of the main characteristics of cases (TB/HIV-infected children) and controls (HIV-infected children). Hospital Pediátrico David Bernardino (HPDB, Angola): January 2005–December 2006.

Variable	Cases *n*	%	Controls *N*	%	Odds ratio	95% CI	*p* value
Age group (months)							
≤36	35	15.2	248	36.9	1.00		
37–60	95	41.3	272	40.4	2.48	1.62–3.78	<0.001
≥61	100	43.5	152	22.6	4.66	3.02–7.20	<0.001
Total	230	100.0	672	100.0			
Sex							
Female	109	47.4	353	52.5	1.00		
Male	121	52.6	319	47.5	1.22	0.90–1.67	0.18
Total	230	100.0	672	100.0			
Orphaned							
No	133	59.1	543	83.1	1.00		
Yes	92	40.9	110	16.9	3.42	2.44–4.78	<0.001
Total	225	100.0	653	100.0			
Sibling died of AIDS							
No	163	91.6	409	97.8	1.00		
Yes	16	8.4	9	2.2	4.18	1.79–9.75	0.001
Total	178	100.0	418	100.0			
Admission point							
Outpatient clinic	74	32.2	450	67.0	1.00		
Hospital	156	67.8	222	33.0	4.27	3.07–5.96	<0.001
Total	230	100.0	672	100.0			
Antiretroviral treatment							
No	54	33.3	76	39.0	1.00		
Yes	108	66.7	119	61.0	1.28	0.83–1.97	0.27
Total	162	100.0	195	100.0			
Mode of HIV transmission							
Vertical	215	95.5	619	94.4	1.00		
Others	10	4.5	37	5.6	0.78	0.38–1.59	0.49
Total	225	100.0	656	100.0			
Immunodeficiency							
Absent	7	3.0	392	58.6	1.00		
Present	222	97.0	277	41.4	44.88	6.73–20.82	<0.001
Total	229	100.0	669	100.0			
Clinical category of HIV (CDC)^*∗*^							
Categories N and A	55	24.0	457	68.1	1.00		
Categories B and C	174	76.0	214	31.9	6.76	4.79–9.53	<0.001
Total	229	100.0	671	100.0			

^*∗*^Reference [20].

**Table 2:** Odds ratios and effectiveness of the association between BCG vaccination and TB according to different diagnostic categories. Hospital Pediátrico David Bernardino (HPDB, Angola): January 2005–December 2006.

Variable	Tuberculosis (all diagnostic categories)		Control		OR (95% CI)	*p* value	(1-OR) (%) (95% CI)
	*N*	%	*N*	%			
BCG vaccination							
No	86	37.4	239	35.6	1.00		
Yes	144	62.6	433	64.4	0.92 (0.68–1.26)	0.62	8 (−26–32)
Total	230	25.5	672	74.5			

Variable	Tuberculosis (confirmed diagnosis)		Control		OR (95% CI)	*p* value	(1-OR) (%) (95% CI)
	*N*	%	*N*	%			

BCG vaccination							
No	20	35.7	239	35.6	1.00		
Yes	36	64.3	433	64.4	0.99 (0.54–1.82)	0.90	1 (−82%–46%)
Total	56	7.7	672	92.3			

Variable	Tuberculosis (probable diagnosis)		Control		OR (95% CI)	*p* value	(1-OR) (%) (95% CI)
	*N*	%	*N*	%			

BCG vaccination							
No	66	37.9	239	35.6	1.00		
Yes	108	62.1	433	64.4	0.90 (0.63–1.29)	0.62	10 (−29–37)
Total	174	20.6	672	79.4			

BCG: Bacille Calmette-Guérin; TB: tuberculosis; OR: odds ratio; CI: confidence interval; (1-OR) (%): BCG effectiveness.

**Table 3:** Odds ratios of the association between BCG vaccination and TB adjusted for biological, family structure, healthcare, and socioeconomic variables and clinical conditions. Hospital Pediátrico David Bernardino (HPDB, Angola): January 2005–December 2006.

Variables	OR_adjusted_	95% CI	*p* value
BCG (adjusted for biological variables)^*∗*^	0.88	0.64 to 1.21	0.42
BCG (adjusted for family structure variables)^*∗∗*^	0.85	0.57 to 1.26	0.41
BCG (adjusted for healthcare variables)^*∗∗∗*^	0.79	0.40 to 1.56	0.50
BCG (adjusted for socioeconomic variables)^*∗∗∗∗*^	0.91	0.66 to 1.27	0.59
BCG (adjusted for clinical condition variables)^*∗∗∗∗∗*^	1.14	0.78 to 1.65	0.49

BCG: Bacille Calmette-Guérin; TB: tuberculosis; OR: odds ratio; CI: confidence interval; ^*∗*^age; ^*∗∗*^age, orphaned, and sibling died of AIDS; ^*∗∗∗*^age, admission point, adherence to antiretroviral treatment, distance to health center, and regularity of AIDS consultations; ^*∗∗∗∗*^age, employment status, number of people living in household, and housing conditions; ^*∗∗∗∗∗*^age, previous history of pneumonia, clinical category of HIV infection (CDC), and degree of immunodeficiency.

**Table 4:** Crude and adjusted effectiveness based on the presence of BCG scar for all children; crude effectiveness based on the vaccination card and BCG scar for those children with a vaccination card. Hospital Pediátrico David Bernardino (HPDB, Angola): January 2005–December 2006.

	OR (95% CI)		(1-OR) (%) (95% CI)	
	Crude	Adjusted^*∗*^	Crude	Adjusted^*∗*^
All children based on BCG scar (*n* = 902)	0.92 (0.68–1.26)	0.70 (0.28–1.75)	8 (−26–32)	30 (−75–72)
Children with vaccination card (*n* = 541)				
Based on the vaccination card	0.71 (0.44–1.14)	—	29 (−14–56)	—
Based on the BCG scar	0.84 (0.53–1.32)	—	16 (−32–47)	—

^*∗*^Adjusted for sibling died of AIDS, regularity of AIDS consultations, adherence to antiretroviral treatment, degree of immunodeficiency, and clinical category of HIV infection (CDC); variables that remained statistically significant in the intrablock multivariable analysis.

BCG: Bacille Calmette-Guérin; CI: confidence interval; OR: odds ratio; (1-OR) (%): effectiveness.

**Table 5:** Odds ratios and effectiveness of the association between BCG vaccination and TB (pulmonary and extrapulmonary). Hospital Pediátrico David Bernardino (Angola): January 2005–December 2006.

Variable	Pulmonary tuberculosis		Control		OR (95% CI)	*p* value	(1-OR) (%) (95% CI)
	*N*	%	*N*	%			
BCG vaccine							
Yes	124	62.0	433	64.4	0.90 (0.64–1.26)	0.58	10 (−26%–36%)
No	76	38.0	239	35.6	1.00		
Total	200	22.9	672	77.1			

Variable	Extrapulmonary tuberculosis		Control		OR (95% CI)	*p* value	(1-OR) (%) (95% CI)
	*N*	%	*N*	%			

BCG vaccination							
Yes	20	66.7	433	64.4	1.10 (0.48–2.57)	0.96	−10 (−157–52)
No	10	33.3	239	35.6	1.00		
Total	30	4.3	672	95.7			

BCG: Bacille Calmette-Guérin; TB: tuberculosis; OR: odds ratio; CI: confidence interval; (1-OR) (%): effectiveness.
