# Supplementary material for: Effectiveness of the First Dose of BCG against Tuberculosis among HIV-Infected, Predominantly Immunodeficient Children
Source: Biomed Res Int. 2015 Jun 29;2015:275029. doi: 10.1155/2015/275029 (PMC4499653; doi:10.1155/2015/275029)
Supplement: Supplementary file 1 — The supplementary material includes the diagnostic criteria for HIV infection in Angola and the techniques used in the study for the CD4 + T cells count, tuberculin test and Acid-Fast Bacilli (AFB) smear and culture. [file 275029.f1.doc]

**Diagnostic criteria and exams**

**Diagnostic exams**

**HIV infection**

The diagnosis of HIV infection was performed in accordance with the standards of the Ministry of Health of Angola [2], using two successive rapid tests, Determine ® (Abbott Diagnostics, USA) and UniGold® (Trinity Biotech, Ireland). If the first was negative, the individual was considered uninfected; if the two were positive the individual was considered infected and if the tests were discordant HIV status was determined by performing Western blot. [(Gaifar®, Potsdam, Germany).

Reference:

1. Van-Dunem J, Fernandes CA, Ferraz D*, et al.* **Avaliação laboratorial do desempenho de diferentes testes rápidos e simples para detecção de anticorpos contra VIH-1/VIH-2 em Angola.** *Acta Médica Angolana* 2004,142:87 - 97.

**Count of T lymphocytes subpopulations**

The count of CD4 + T cells per mm^3^ and their percentages were determined by flow cytometry (FACSort®, Becton Dickinson, San Jose, CA, USA), using whole blood. The degree of immune deficiency was defined according to the revised criteria of the Centers for Disease Control and Prevention (CDC) [3].

Reference:

1. Centers for Disease Control and Prevention. **Revised classification system for human immunodeficiency virus infection in children less than 13 years of age**. *Morb Mortal Wkly Rep* 1994,43:1 - 10.

**Tuberculin test**

Tuberculin test was performed using the Mantoux technique and followed the WHO recommendations. The 2UT PPD-RT 23 / ​​0.1 ml (Statens Serum Institute, Copenhagen, Denmark) was used and the induration was read 72 hours after administration, by trained nurses, blinded to the research objective.

**Smear and Culture for *Mycobacterium tuberculosis***

Smear and culture were performed in the National Institute of Public Health. The material, in cases of suspected pulmonary tuberculosis, was obtained from gastric aspirates (in children aged less than or equal to five years) and from spontaneous or encouraged expectoration (in children older than five years).

The collection of samples was performed on three successive days, in the morning, after overnight fasting. For the collection of other secretions and body fluids, only one sample for culture was required. The material was added to a saline phosphate solution, preserved in airtight container at a temperature of ± 4° C for a period not exceeding 48 hours, when it was not possible to immediately analyze the material. Ziehl-Neelsen staining and culture in Lowenstein-Jensen solid medium were used. The incubation period for the cultures was eight weeks.
